# Supplementary material for: Increased thermal suitability elevates the risk of dengue transmission across the mid hills of Nepal
Source: PLoS One. 2025 Apr 24;20(4):e0322031. doi: 10.1371/journal.pone.0322031 (PMC12021130; doi:10.1371/journal.pone.0322031)
Supplement: Attachment — Submitted filename: Response to reviewers_jan30.pdf [file pone.0322031.s003.pdf]

Reviewer #1

This study was utilized Moderate Resolution Imaging Spectroradiometer satellite land surface temperature data (MOD11A2) and a temperature dependent mechanistic model to predict the monthly suitability and its changing pattern for dengue transmission in Nepal from 2000 to 2020 for both mosquito vectors, Aedes aegypti and Ae. albopictus. This study estimated the population at risk of dengue based on different lengths of the transmission season. The results from this study could be a model for dengue management and vectors control in Nepal and other dengue endemic countries.

Response:

Dear reviewer,

Thank you very much for a detailed review of the manuscript and providing valuable suggestions for its improvement. We have followed your suggestions on revising the manuscript and also made point-by-point responses to all the comments.

However, there are some comments for this manuscript to address as follows:

1. Citations of the references should follow the author guidelines of PLOS ONE. Most of the citations presented as author name and year, but some citations presented as the number. For examples, the references on Page 9, Lines 195-196, "…………including sea surface temperature reconstruction [55–57] ,………, on Page 10, Lines 230 and 233………the calculation and interpretation of R0 [24–28]. and …………equilibrium prevalence [29–31]." Please check the citation of references according to the author guidelines of PLOS ONE.

Response: Thank you very much for pointing this error. We have revised all the citations and references according to the guidelines of PLOS ONE.

2. The term "dengue fever" and "dengue" have different meaning. The meaning of "dengue fever" is the degree of disease severity whereas "dengue" stands for disease occurring by dengue viral infection. Please revise the following sentences and other sentences.

a. On Page 3, Lines 61-62, "Currently, there are no effective vaccines or specific therapies available to curb the rapid global spread of dengue fever (Simon-Lorière et al., 2017).

b. On Page 5, Line 139, "This study aimed to assess the thermal suitability for dengue fever in Nepal and analyze………."

Response: We apologize for this error. We agree to you on this matter of dengue and dengue fever. We have made necessary corrections.

3. The introduction part should be shorten, concise, and has supporting information relevant to the rational of the study. For example, "A land surface temperature (LST) as low as 13.8 °C in winter has been identified as critical for Ae. aegypti larvae, potentially leading to their near disappearance in subtropical regions of Taiwan during the East Asian winter monsoon (Tsai et al., 2018) on Page 3, Lines 80-82." and "An LST of 13.8 °C in winter has been identified as critical for the survival of Ae. aegypti larvae (Tsai et al.,2018)." On Page 4, Lines 87-89. These two sentences have similar meaning.

Response: Thank you very much for this suggestion. We have shortened the introduction section retaining the intended message.

4. Figure 1 has four major physiographic zones, but Siwalik and Tarai have the same number of 4. However, on Page 6, Lines 152-154, "Nepal is divided into five major physiographic zones- Tarai (below 600 m), Siwalik (100–2000 m), Hill (200–3500 m), Middle Mountain (700–4100 m) and High Mountain (1800–8800 m) (LRMP, 1986)."

Response: Sorry for this error in the legend of figure 1. Tarai should have been labeled as 5. We have corrected it.

5. Materials and Methods :

a. Page 8, Lines 173-174, "………(R Development Core Team)", the statistical program R should be cited correctly.

Response: Thank you. We have updated the citation.

b. Page 8, Line 179, "………we extracted TIF layers from originally supplied HDF file, …." The abbreviation for file type should be cited as full name, then followed by abbreviation.

Response: Thank you for this suggestion. We have added the full names for those abbreviations.

c. Page 8, Lines 174-178, "The MOD11A2 is 8-day averaged LST product derived from MOD11A1 (Z. Wan, 2015). The MODA1 is daily LST product derived by computation from two adjacent thermal infrared bands, 31 and 32. The product contains both day and night-time surface temperature bands and their quality indicator (QC) layers. MOD13A2 includes one LST of day and one LST of night which in some sense represent the maximum and minimum temperature of the 24-hour." Which The Moderate Resolution Imaging Spectroradiometer satellite land surface temperature data (MOD11A2, MOD11A1, MOD13A2) was used in this study? These are confusing to the readers.

Response: We used MOD11A2 for extracting the temperature variables and downstream analyses. We included the description of the data generation and processing by the satellite sensors. These information are available in the data source (https://lpdaac.usgs.gov) which is cited in the manuscript. Hence, we removed this description from the manuscript.

d. Page 9, Lines 208-209, ……….entire study period into two phases, 2000–2010 (the period of dengue emergence in Nepal) and 2010–2020 (the period of rapid expansion in Nepal)." and Table 1. The study period are divided into two phases, but the time frame for each period are over-lapping. Please state clearly.

Response: We apologize for this error. We have analyzed data from the beginning of 2001 to the end of 2020. Therefore, it has been updated accordingly. "………………entire study period into two phases, 2001–2010 (the period of dengue emergence in Nepal) and 2011–2020 (the period of rapid expansion in Nepal)"

e. Page 10, Line 224, the formula and descriptions of each parameters should be precise. These are confusing to the readers.

Response: Thank you. We have revised it and tried making it clearer to the readers.

6. Results :

a. Page 15, Line 327, "…………….observed in" Please check.

Response: Sorry for the incomplete sentence. We have added the missing information to the sentence.

b. All values in percentage should be systematic, i.e., one or two decimal points.

Response: Thank you for this suggestion. We have maintained two decimal points for the percentage throughout the manuscript.

c. Page 16, Lines 355-356, "This proportion was lower in 2010; 88.76% for Ae. aegypti mosquito and 93.43% Ae. albopictus." But Table 2 show these data in the year 2000. Please check.

Response: We apologize for this error. It should have been 2010, not 2000. We have corrected it.

7. Discussion : Please add limitation of the study.

Response: Thank you for this suggestion. The last paragraph of the discussion section discusses about the empirical and methodological limitations of this study. Please refer to the last paragraph of the revised manuscript.

These are all issues raised for this manuscript and major revision is needed.

Response: Thank you very much for these important suggestions and corrections. We appreciate your contributions for the improvement of the manuscript.

Reviewer #2:

An interesting project and well-written manuscript. Just a few points to note and include:

Response:

Dear reviewer,

Thank you for the comments and suggestions for improvement of the manuscript. We have revised it following your suggestions and also made point-by-point responses as follows:

1. typing error - Line 465

Response: Thank you. We have rectified it.

2. Can include discussion the limitations of DINEOF method.

Response: Thank you for this suggestion. We have added the limitation of the study including that of DINEOF method on the last paragraph of the discussion.

3. Can include introduction in the role of microclimate on the development of both vector species.

Response: Thank you very much for this suggestion. Temperature and humidity are the two major microclimatic conditions affecting the development of *Aedes* species. We have discussed them in the background section.

4. Impacts of other meteorological factors other than temperature.

Response: Meteorological factors other than temperature might also have impacts on dengue transmission. Therefore, we have discussed this in the limitation section of the discussion and suggested to consider other

5. Specific suggestions for the health authority to control dengue transmission.

Response: Thank you for this suggestion. We have added following recommendations at the end of the conclusion section- "Dengue control and management within Nepal should be improved through evidence based interventions by focusing on regions at elevations between 500–1500 m and urban centers which have the most significant growth of thermal suitability and population exposure risk to dengue transmission within the past two decades."
