# Supplementary material for: Increased thermal suitability elevates the risk of dengue transmission across the mid hills of Nepal
Source: PLoS One. 2025 Apr 24;20(4):e0322031. doi: 10.1371/journal.pone.0322031 (PMC12021130; doi:10.1371/journal.pone.0322031)
Supplement: Attachment — Submitted filename: Reponse to Reviewers_11 March.pdf [file pone.0322031.s004.pdf]

Reviewer #1: All issues raised by reviewers have been addressed. Interestingly, this study could be a model for dengue management and vectors control in dengue endemic countries.

Response: Dear reviewer, thank you for your positive comments on the manuscript. We appreciate constructive comments and suggestions in the previous round of review.

Reviewer #3: - Overall, this manuscript has shown excellent scientific work, covering the data from 2001 to 2020. However, I have some minor concerns to improve its quality. Here are my comments:

- (page 3, lines 55-57) this statement, “It has been a leading public health challenge worldwide causing approximately 390 million ...”, also shares similar ideas to a study by Masyeni et al. entitled “Cytokine profilesin dengue fever and dengue hemorrhagic fever: A study from Indonesia”. Kindly cite this article to strengthen the statement.

Reply: Thank you so much for your comment. We found that the suggested literature is concerned with cytokine dysregulation in dengue fever and is a study from Indonesia alone. It does not accommodate our statement of dengue as a public health challenge worldwide. The literature we have cited for this statement are of global coverage and authentic. Therefore, unfortunately, we opted not to cite the suggested literature.

- (page 17, lines 370-371) I suggest that authors rewrite this sentence into "Elevation is a key determinant of the spatial distribution of temperature-sensitive arboviral diseases." to make it clearer.

Reply: Thank you for this suggestion. We have revised the sentence as you suggested.

- (page 3, lines 70-71) this sentence “However, thermal extremes can negatively impact disease dynamics…” will be more significant if it’s also supported by another relevant study. For example, Ahmad K and Chiari W. Metal oxide/chitosan composite for organic pollutants removal: A comprehensive review with bibliometric analysis.

Reply: Thank you so much for your comments.

The suggested paper focusing on organic pollutants removal is irrelevant with this very manuscript focusing on the effects of environmental temperature rise on dengue transmission. Therefore, we could not cite it.
